# Selective strategic adaptation and structural trade-offs in a public traditional Chinese medicine hospital under China's national performance appraisal: a longitudinal study

**DOI:** 10.3389/frhs.2026.1809377

**Published:** 2026-05-08

**Authors:** Xiao Du, Changchang He, Dongna Zhang, Zifan Wang, Mingyu Zhao

**Affiliations:** 1 Department of Healthcare Quality and Safety, Luoyang Orthopedic-Traumatological Hospital of Henan Province (Henan Provincial Orthopedic Hospital), Luoyang, China; 2 Medical Physics Group, Department of Biomedical Imaging, Faculty of Medicine, University of Malaya, Kuala Lumpur, Malaysia

**Keywords:** DRG/DIP payment reform, grey relational analysis, high-quality development, human capital investment, national performance appraisal, public hospital reform, strategic adaptation, traditional Chinese medicine hospital

## Abstract

**Background:**

Since 2019, China's National Performance Appraisal (NPA) has guided tertiary public hospitals from scale-oriented expansion toward high-quality development. While its impact on general hospitals has been widely discussed, the adaptation strategies of traditional Chinese medicine (TCM) hospitals, which face the dual constraints of modernization and traditional preservation, remain underexplored. This study draws on institutional theory, resource dependence theory, and agency theory to evaluate the longitudinal impact of the NPA on a Grade-A tertiary TCM orthopedic hospital during the post-pandemic recovery period.

**Methods:**

A single-center retrospective observational study was conducted using longitudinal data from 2019 to 2024 and selected key performance indicators across four NPA dimensions: medical quality, efficiency, sustainability, and satisfaction. Grey relational analysis was used to identify weighted drivers of development, while Spearman's rank correlation analysis was used to assess the potential crowding-out relationship in resource allocation.

**Results:**

The hospital demonstrated selective strategic adaptation under layered reform pressures. While core clinical safety indicators remained stable and competitive, several high-weight indicators showed a marked 2024 step-change. TCM decoction piece usage rose to 17.74% in 2024, moving from consistently below the annual national peer median during 2019–2023 to slightly above the 2024 median (17.74% vs. 16.49%), suggesting threshold-crossing catch-up with strategic differentiation. Research funding per 100 health professionals also increased by 115%. Together, these changes likely reflected the convergence of post-pandemic recovery, strategic realignment under the NPA, and broader reform pressures. Spearman analysis showed a moderate inverse association between the debt-to-asset ratio and the personnel expenditure ratio $\left(r_{s}=-0.486,\;p=0.329\right)$, suggesting a crowding-out tendency, although the limited number of annual observations precludes strong statistical inference.

**Conclusion:**

The NPA appears to have contributed to a strategic shift toward research intensity and stronger TCM-characteristic performance, but the 2024 acceleration should be interpreted within the combined context of post-pandemic recovery and layered reform pressures. This apparent success remains financially precarious because it relies on a structural trade-off in which high leverage coexists with persistently constrained personnel investment. Sustainable development will require policy adjustments that better balance performance scoring, financial resilience, and human capital investment.

## 1 Introduction

Over the past two decades, China's healthcare system has undergone a profound paradigm shift, oscillating between market-oriented deregulation and centralized government control, striving to find the optimal equilibrium between efficiency and equity (1). Following the landmark “New Healthcare Reform” launched in 2009, its main policy objectives focused on expanding healthcare coverage and improving affordability (basic medical insurance) (2). This initial phase achieved significant results, ultimately accomplishing near-universal medical insurance coverage that encompasses over 95% of the population, while also substantially reducing patients' out-of-pocket expenses (3). However, the rapid expansion of insurance coverage and the fee-for-service payment model triggered an unintended structural consequence: it intensified the fierce “bed expansion race” among tertiary public hospitals (4, 5).

Between 2010 and 2018, the total number of beds in China's tertiary public hospitals nearly doubled, creating some of the world's largest healthcare complexes. This rapid growth was driven by a revenue model oriented toward volume rather than value (6). Hospitals heavily relied on drug price markups (until the implementation of a zero-markup policy in 2017), high-volume diagnostic services, and the purchase of capital-intensive equipment such as high-end CT and MRI scanners to maximize revenue (5, 6). While this growth model effectively increased the aggregate capacity of services and shortened waiting times, it also led to soaring national healthcare costs, systemic inefficiencies, and growing public concerns about the quality and safety of healthcare (6). This phenomenon, which critics call “industrialized healthcare,” made patient volume the prioritized, or even sole, measure of an institution's success, while neglecting patient outcomes and service quality (7).

To center efforts on alleviating these deep-seated structural inefficiencies and aligning public hospitals back with their fundamental public welfare mission, the General Office of the State Council promulgated the “Opinions on Strengthening the Performance Appraisal of Tertiary Public Hospitals” (State Council Document No. 4 [2019]) in January 2019 (8). This key policy introduced the National Performance Appraisal (NPA), a system hailed in the healthcare industry as the baton or gold standard of health reform. It marks a paradigm shift in healthcare governance from a volume-oriented incentive mechanism to a value-oriented governance mechanism (9). Unlike previous evaluation systems that prioritized gross revenue, number of beds, and scale, the NPA explicitly focuses on high-quality development (10). It establishes a standardized, data-driven framework that evaluates hospitals across four major dimensions: medical quality, operational efficiency, sustainable development, and satisfaction, and comprises 34 specific national monitoring indicators for traditional Chinese medicine (TCM) hospitals (11).

The NPA transcends the function of a simple ranking system; it has become a high-stakes governance instrument with significant administrative influence. The results are directly linked to subsidies from central and local governments, hospital accreditation levels (e.g., maintaining a Grade-A hospital status), approvals for new bed quotas and equipment purchases, and, crucially, the appointment, dismissal, or promotion of senior hospital management (8, 12). Therefore, the NPA constitutes a hard constraint on hospital administrators, urging them to transit from scale expansion to connotation-oriented development. It compels hospital leaders to shift their strategic focus from building new facilities to optimizing clinical pathways, improving diagnostic accuracy, and promoting scientific research innovation (13).

In this far-reaching wave of transformative reforms, TCM hospitals occupy a unique and often challenging institutional position. Unlike general hospitals that follow a relatively linear path of biomedical modernization, TCM institutions are forced to confront a unique dual identity dilemma (14). This dilemma gives rise to a series of complex operational contradictions and strategic trade-offs, complicating strategic planning (15). Unlike general hospitals where modernization and efficiency often align, TCM hospitals face a unique conflict where NPA-driven modernization (capital-intensive) competes directly with TCM-characteristic mandates (labor-intensive). Because the NPA simultaneously rewards electronic medical record (EMR) capability, research output, and other modernization-related indicators while also assigning substantial weight to decoction and non-drug therapy indicators, it institutionalizes a resource conflict between capital deepening and labor-dependent TCM service delivery (11, 16, 17).

First, TCM hospitals face an urgent need for modernization. To remain competitive in a Western-dominated healthcare market and ensure patient safety standards comply with international norms, TCM hospitals must adopt the identical modern medical standards as general hospitals (14, 18). This includes integrating advanced surgical techniques (e.g., robotic-assisted orthopedics, minimally invasive surgery), adopting Level 5 or higher EMR systems for data interoperability and clinical decision support, and strictly implementing antimicrobial stewardship programmes, etc (11, 15, 19, 20). Patients and regulatory agencies expect tertiary TCM hospitals to possess the same diagnostic capabilities as top-tier medical centers, such as 3.0T MRI, 64-slice CT, and automated laboratory systems. Without these capabilities, TCM hospitals risk being marginalized, relegated to supplementary care institutions rather than primary treatment centers for acute and complex diseases (21, 22).

Second, there are constraints imposed by traditionalism. The NPA has established specific TCM-exclusive indicators designed to prevent the Westernization of TCM institutions (11). This concern stems from observations that many TCM hospitals, in their pursuit of modernization, have gradually lost their distinctive treatment characteristics, becoming overly reliant on Western medicine and surgery (23, 24). To curb this trend, the NPA administers penalties on TCM hospitals that fail to maintain a high ratio of non-drug therapies (e.g., acupuncture, tuina, cupping, moxibustion) and herbal decoctions (25). Moreover, indicators related to TCM pharmaceuticals and non-pharmaceutical therapies are varied and carry high weightings (11). This policy aims to safeguard the cultural and clinical heritage of TCM, but it also imposes rigorous operational restrictions, which may conflict with the efficiency goals.

This inherent duality creates significant tension in resource allocation. Modernization is imperative, but it is inherently capital-intensive, requiring substantial and continuous investment in imported hardware, advanced diagnostic equipment, and software infrastructure. In stark contrast, traditional Chinese medicine treatments are labor-intensive and time-consuming, relying primarily on the skilled manual techniques of practitioners rather than automated instruments and, in many settings, yield a service mix that is more labor-dependent and less easily scalable than Western medicine departments (15–17, 26–28). For example, while robotic surgery can generate high revenue, its consumable costs are also high; while a course of acupuncture treatment may generate lower revenue, its labor input is high. How TCM hospitals can deal with the financial demands of modern infrastructure and policy mandates for traditional characteristics remains a crucial but underexplored issue in the field of health policy. The related capital-labor tension is central to the present study because the hospital's strategic adaptation is evaluated not only by its output gains but also by whether those gains are achieved through a financially fragile internal allocation pattern. More recent literature suggests that the NPA should be understood within the broader second phase of China's public hospital reform, in which “high-quality development” has become the dominant policy orientation since 2021. In this phase, performance governance, digital modernization, specialty development, and payment reform are increasingly intertwined rather than operating as separate policy streams (29–31). This broader reform context is particularly relevant for TCM hospitals because it intensifies the simultaneous pressure to strengthen both modern governance capacity and TCM-characteristic service delivery.

To analyze the behavioral adaptation of TCM hospitals under the NPA, this study integrates three theoretical frameworks from organizational sociology and economics: institutional theory, resource dependence theory, and agency theory.

First, the theory of institutional isomorphism posits that organizations in highly structured domains tend to homogenize to ensure their legitimacy (32). In the context of the NPA, this process manifests itself through three mechanisms: 1) Coercive isomorphism: Driven by regulatory pressure, hospitals initially adopted compliance behavior to meet minimum standards and avoid administrative penalties; 2) Mimetic isomorphism: In situations of environmental uncertainty (e.g., the COVID-19 pandemic), organizations emulate successful peers to mitigate risk; 3) Strategic isomorphism: This describes a nuanced evolution process in which hospitals shift from passive compliance to proactively optimizing behavior (32–34). They may aggressively reallocate resources to high-weighted performance indicators to maximize ranking scores.

Second, resource dependence theory (RDT) argues that organizations depend on their environment for access to critical resources (35). For public hospitals in China, resources include not only revenue, but also political legitimacy gained through the NPA rankings that determine governmental subsidies, accreditation, and policy benefits (36). RDT suggests that hospitals will fundamentally alter their internal structures to satisfy the government, which is the primary controller of these critical resources, to secure survival and growth (37).

Third, agency theory provides a framework to examine the relationship between the principal (government) and the agent (hospital administrator) (38). While the government prioritizes public welfare, cost control, and quality of care, administrators aim for revenue maximization, reputation, and career advancement (39). The NPA serves as a contract that aligns these interests. Due to information asymmetry, agents may engage in “signaling” behavior by focusing on improving observable metrics such as research funding and TCM usage to score points, but this may come at the expense of unobservable holistic, long-term outcomes (40, 41).

## 2 Materials and methods

### 2.1 Study design

This study employs a single-center, observational, retrospective longitudinal design. The subject is a representative Grade-A tertiary public TCM orthopedic hospital in central China.

While annual national reports provide aggregated data on the NPA trends, they typically present a macro perspective, obscuring the micro-level strategic behavior of individual institutions (42). Existing literature is predominantly cross-sectional or short-term, lacking longitudinal, single-center studies that capture dynamic organizational adjustment processes (43–46). Consequently, this study involves: 1) Temporal relevance: Utilizing post-pandemic data to reveal strategic shifts following the normalization of hospital operations. Most current studies only cover data up to 2022 (45, 46), missing the critical period of post-pandemic strategic adjustment, in which strategic transformation, recalibrating strategies once acute disruptions subside, is most likely to occur; 2) Structural analysis: Quantitatively measuring whether pursuing high NPA scores (through capital-intensive investment) comes at the expense of financial health or employee compensation. Few studies directly link multifaceted indicators with financial solvency indicators in a single model to explore the potential trade-offs; 3) Methodological depth: Employing advanced systems modeling methods like grey relational analysis (GRA) to rank the driving factors of hospital development in the context of TCM. This approach goes beyond simple correlation analysis to identify weighted influences, specifically addressing the challenge of handling real-world, non-linear hospital management data.

This study addresses these gaps by analyzing the performance trajectory of the target hospital from 2019 to 2024. Applying Spearman's rank correlation and GRA, it aims to empirically examine whether the hospital has shifted from initial compliance toward selective strategic adaptation under the NPA. Furthermore, the study seeks to quantify the structural financial costs associated with this transition, assessing whether performance-oriented development has created internal resource allocation tensions. Given the limited number of annual observations (N=6), the correlation analysis was intended to identify indicative structural trends rather than definitive causal laws.

#### 2.1.1 Rationale for selection

As a designated National Regional Medical Center and a Grade-A tertiary TCM hospital, this institution represents the upper tier of China's public hospital system. Its specialized nature (orthopedics) makes it particularly sensitive to both regulations on high-value consumables (national volume-based procurement of implants) and requirements of TCM characteristics (47). It has great potential to serve as a stress test case for examining the impact of the NPA. Moreover, as a provincial hospital with its main campus located in a non-capital, prefecture-level city, it faces dual competitive pressure from national medical centers in Beijing or Shanghai and local municipal hospitals, making its strategic choices highly reflect the squeezed middle dilemma within the hospital system.

#### 2.1.2 Study period

The study covers six full fiscal years from January 1, 2019, to December 31, 2024. This period is strategically selected to span the pre-pandemic baseline, the operational disruptions during the COVID-19 pandemic (2020–2022), and the critical post-pandemic strategic adjustment phase (2023–2024).

### 2.2 Data collection

#### 2.2.1 Data source and sample selection

The data were collected from hospital systems such as EMR and financial statements, constituting the records of the hospital's daily operations and healthcare practices. The data are reported to the “National Tertiary Public TCM Hospital Performance Monitoring System” and audited by the National Health Commission (NHC) on a routine basis. The use of data is compliant with the relevant regulations of the national system (48, 49). The study only collected de-identified administrative data.

The following table (Table 1) presents the annual sample volumes and core operational metrics of the studied hospital, providing the fundamental quantitative baseline for the entire longitudinal analysis throughout the study period.

**Table 1:** Annual sample size and core operational baseline (2019–2024).

Metric	2019	2020	2021	2022	2023	2024
Discharged patient volume	81,158	85,429	91,534	88,217	95,843	103,726
Outpatient volume	531,601	553,735	672,171	554,918	605,163	670,978
Number of health professionals	1,990	2,034	2,327	2,537	2,651	2,709
Number of surgical cases	31,821	32,463	39,013	36,784	49,330	54,216
Number of Class I incision surgical cases	24,184	24,347	30,041	26,852	39,957	42,831
Total bed days for discharged patients	1,209,254	1,307,064	1,391,317	1,393,828	1,271,066	1,412,120

#### 2.2.2 Indicator system

The study focused on ten national monitoring indicators that were selected based on their weight within the evaluation system and their relevance to the hypotheses of the study (Table 2). These indicators are categorized into the four standardized dimensions (medical quality, efficiency, sustainability, and satisfaction) of the NPA framework (11). The indicators of TCM characteristics are listed separately due to their unique impact. The NHC provides the “national peer median” for each indicator every year. To create a robust benchmark, the study calculated the average of these annual medians across the study period. This enabled a gap analysis, effectively contextualizing the hospital's performance against comparable institutions nationwide.

**Table 2:** Key performance indicators (KPIs) selected for analysis.

Dimension	Key Indicators	Rationale for Selection
Medical quality	Class I incision infection rate	Measures patient safety, clinical effectiveness, and the level of digital modernization. These are “foundational indicators” where failure results in immediate disqualification or severe penalties.
	Antibiotic use intensity (DDDs)	
	Electronic medical record (EMR) level	
	Surgical complication rate	
Operational efficiency	Debt-to-asset ratio	Measures financial health and resource allocation rationality. These are “constraint indicators” that limit the scope of expansion.
	Personnel expenditure ratio	
Sustainable development	Research funding per 100 health professionals	Measures innovation capability and academic output. This is a “bonus indicator” that differentiates top-tier hospitals from average hospitals.
TCM characteristics	Percentage of TCM decoction piece usage	Measures the degree of adherence to the core TCM identity and the compliance with cultural mandates. These are “identity indicators” unique to TCM hospitals.
	Percentage of outpatient TCM non-drug therapy	
Satisfaction	Patient satisfaction (weighted score for outpatient & inpatient services)	Measures the patients’ perception of the quality of outpatient and inpatient care across the service journey. This is a perception-based “outcome indicator” that reflects hospitals’ service responsiveness and patient-centeredness.

DDDs, defined daily doses.

### 2.3 Statistical analysis strategies

Given the complexity and non-linear nature of hospital management systems, as well as the limited time series (N=6), traditional regression analysis, which typically assumes large samples and distributional stability, is insufficient and may even be misleading (50). In the preliminary analysis, Pearson correlation was explored. However, because linearity, joint normality, and outlier influence could not be reliably assessed with only six annual observations, the current study adopted Spearman's rank correlation as the primary correlation method. Spearman's coefficient is a non-parametric measure of monotonic association and is more appropriate when distributional assumptions cannot be confidently verified (51, 52). Therefore, the study adopted a hybrid methodological approach that combines Spearman's rank correlation for structural trend assessment and grey relational analysis for driver identification (53, 54). A Spearman correlation matrix was also constructed to visualize the pairwise associations among key structural variables.

#### 2.3.1 Spearman rank correlation and sensitivity analysis of the crowding-out hypothesis

To empirically test the trade-off hypothesis, the Spearman rank correlation coefficient $\left(r_{s}\right)$ between financial leverage and human capital investment was analyzed. The debt-to-asset ratio was used as a proxy for financial leverage, whereas the personnel expenditure ratio was used as a proxy for human capital investment.

Hypothesis *H*_1_: There exists an inverse monotonic association between leverage and personnel spending. If present, this pattern would suggest that higher debt servicing pressure may crowd out the fiscal space available for wage growth.

Because the study contained only six annual observations, the correlation coefficients were interpreted as indicative of trends rather than definitive causal laws. The Spearman analysis therefore served both as the primary non-parametric test and as a sensitivity-oriented robustness check against the assumption-dependent Pearson coefficient in a small time series (50–52). Accordingly, the correlation results were used to characterize the direction and relative strength of structural association, not to establish causation.

All statistical analyses were performed using Python 3.9 (Pandas, SciPy) and verified using Microsoft Excel 2019. Spearman coefficients and two-sided *P* values were calculated in SciPy, and the underlying rank ordering was cross-checked manually in Excel 2019.

#### 2.3.2 Grey relational analysis (GRA)

GRA, developed by Deng Ju-Long in grey system theory, is a method specifically designed to analyze systems exhibiting characteristics of partial information and uncertainty (55). It is widely applied and validated in healthcare management literature to identify the primary drivers of a system's trajectory, particularly suitable for scenarios with limited data points and intricate, non-linear underlying relationships between variables (54). Unlike regression analysis, which aims to find the best-fit line, GRA seeks to measure the geometric similarity between a reference sequence and comparison sequences (56). These features made it robust for the single-center longitudinal nature of this study.

Step 1 was reference sequence selection. The study selected “discharged patient volume” (*X*_0_) as the reference sequence. Patient volume serves as the most direct proxy for the overall development scale, service capacity, and social impact of the hospital. Also, it is a neutral, outcome-level proxy that is less subject to policy gaming than revenue or scores. As shown in Table 1, the discharged patient volumes for the years 2019 through 2024 were 81,158, 85,429, 91,534, 88,217, 95,843, and 103,726, respectively.

Step 2 was data normalization. Given that the units for each indicator are disparate (e.g., percentages, Chinese yuan, ratios), the data must be normalized to a dimensionless 0-to-1 interval to ensure comparability (57).

For benefit-type indicators such as research funding and TCM non-drug therapy usage, where higher values are preferred, the study adopted the “upper-bound effectiveness” method (57):

$$x_{i}^{*}(k)=\frac{x_{i}(k)-\min\;x_{i}(k)}{\max x_{i}(k)-\min x_{i}(k)}$$

For cost-type indicators such as infection rate and debt-to-asset ratio, where lower values are preferred, the study adopted the “lower-bound effectiveness” method (57):

$$x_{i}^{*}(k)=\frac{\max\;x_{i}(k)-x_{i}(k)}{\max\;x_{i}(k)-\min x_{i}(k)}$$

Step 3 was grey relational coefficient calculation. The correlation coefficient $\xi_{i}(k)$ between the comparison sequence *X_i_* and reference sequence *X*_0_ at time point *k* is calculated as follows:

$$\xi_{i}(k)=\frac{\mathrm{mi}n_{i}\;\mathrm{mi}n_{k}\;\Delta_{0i}(k)+\rho \;\mathrm{ma}x_{i}\;\mathrm{ma}x_{k}\;\Delta_{0i}(k)}{\Delta_{0i}(k)+\rho \;\mathrm{ma}x_{i}\;\mathrm{ma}x_{k}\;\Delta_{0i}(k)}$$

Here, $\Delta_{0i}(k)=|x_{0}^{*}(k)-x_{i}^{*}(k)|$, represents the absolute difference between the normalized sequences. Additionally, ρ serves as the resolution coefficient. We set ρ=0.5, which is the standard value used in most econometric studies to ensure adequate distinguishability (55).

Step 4 was grey relational grade calculation. The overall grey relational grade, denoted as $\gamma_{i}$, represents the degree of correlation between factor *i* and the reference sequence over the entire time series. It is calculated as the average of the grey relational coefficients:

$$\gamma_{i}=\frac{1}{n}\overset{n}{\underset{k=1}{\sum }}\xi_{i}(k)$$

Where *n* is the number of data points, while $\xi_{i}(k)$ represents the grey relational coefficient at point *k*.

The resulting value of $\gamma_{i}$ reflects the strength of the relationship. $\gamma_{i}>0.8$ indicates a strong driving relationship, $0.5<\gamma_{i}<0.8$ indicates a moderate relationship, and $\gamma_{i}<0.5$ indicates a weak relationship (58).

## 3 Results

### 3.1 Descriptive statistics of key performance indicators

The following table (Table 3) presents six years of longitudinal data for the included indicators, alongside the national peer median for reference.

**Table 3:** Longitudinal comparison of hospital performance vs. national peer median (2019–2024).

Indicator	Metric	2019	2020	2021	2022	2023	2024	National Peer Median (Avg)
I. Medical quality								
Class I incision infection rate	%	0.00	0.00	0.06	0.03	0.01	0.00	0.15
Antibiotic use intensity	DDDs	30.70	28.67	37.76	37.74	37.47	34.50	29.13
Electronic medical record (EMR) level	Level	4	4	4	4	4	4	3.67
Surgical complication rate	%	0.00	0.00	0.00	0.00	0.34	0.02	0.12
II. Operational efficiency								
Debt-to-asset ratio	%	60.10	61.50	62.10	62.33	61.80	61.95	36.92
Personnel expenditure ratio	%	33.70	33.50	33.20	33.10	33.90	35.80	44.15
III. Sustainable development								
Research funding per 100 health professionals	CNY	190,954	155,113	116,458	63,854	324,513	699,575	98,333
IV. TCM characteristics								
Percentage of TCM decoction piece usage	%	6.11	6.32	6.34	6.39	8.50	17.74	15.25
Percentage of outpatient TCM non-drug therapy	%	0.87	3.18	8.33	13.73	14.56	13.53	31.63
V. Satisfaction								
Patient satisfaction (weighted score for outpatient & inpatient services)	Score	86.30	86.35	86.45	87.50	92.40	93.90	92.58

CNY, Chinese yuan.

### 3.2 Longitudinal trends: emergence of a 2024 step-change

**Figure 1:**
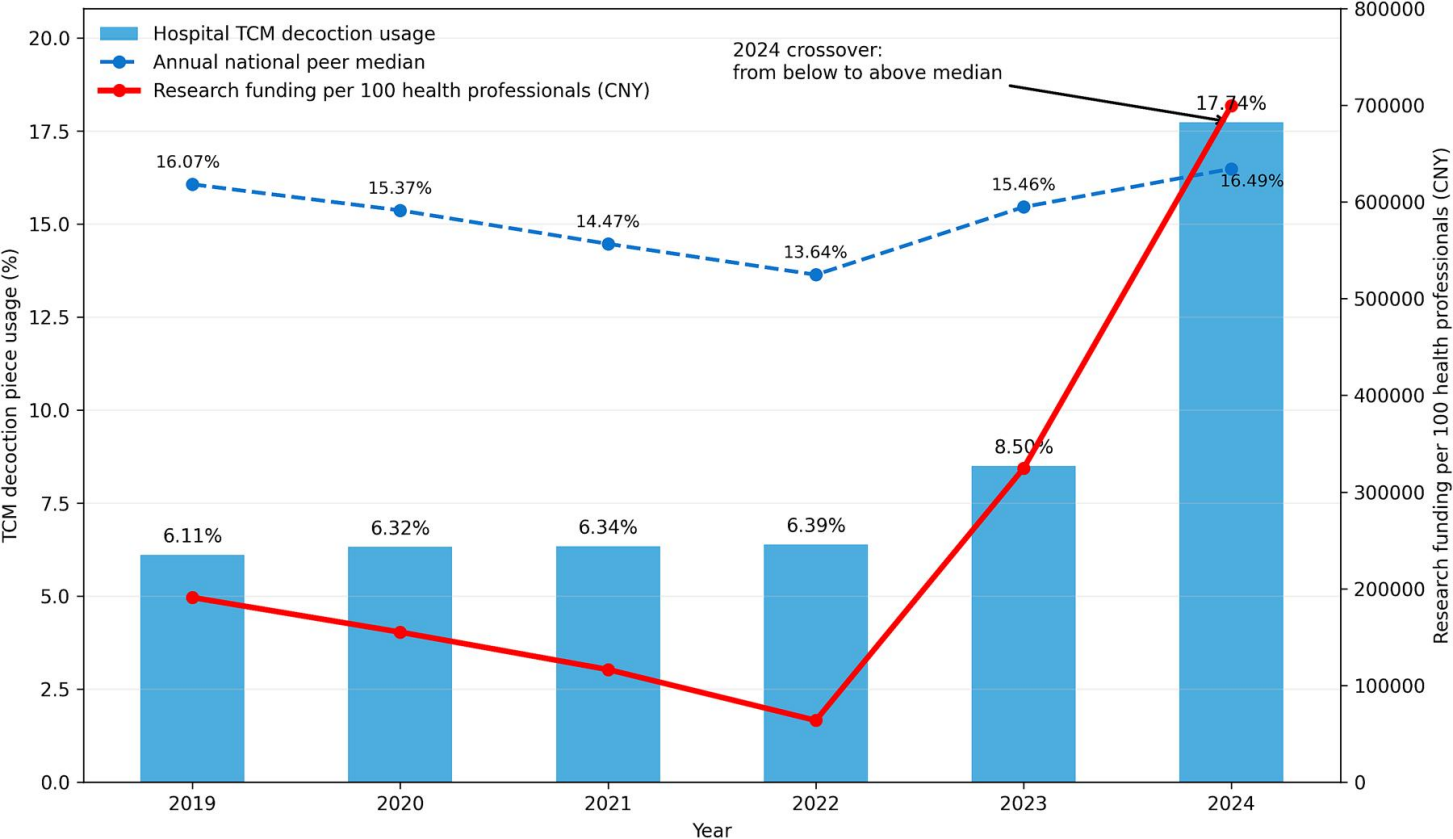
Annual trends in hospital TCM decoction piece usage, its annual national peer median, and research funding per 100 health professionals (2019–2024), showing a 2024 step-change and crossover from below-median to slightly above-median TCM decoction usage.

The most striking finding from the longitudinal data was not a classic J-curve, but rather a marked 2024 discontinuity in selected high-weight indicators. During the initial five years (2019–2023), the hospital largely followed a gradual adjustment path characterized by stability, compliance, and incremental improvement. In 2024, however, several policy-sensitive indicators accelerated discontinuously, suggesting selective intensification rather than uniform convergence (Figure 1).

#### 3.2.1 Rapid expansion of TCM characteristics

To ensure that TCM hospitals retain their distinctive identity and avoid excessive Westernization, the NPA places substantial weight on the use of traditional therapies.

##### 3.2.1.1 Historical trend (2019–2023)

From 2019 to 2023, the proportion of TCM decoction piece (herbal slice) usage remained at a relatively low and stable level, ranging from 6.11% to 8.50%. During the same period, the hospital remained consistently below the annual national peer medians, which were 16.07%, 15.37%, 14.47%, 13.64%, 15.46%, respectively. This pattern suggests that, prior to 2024, the hospital had not yet fully aligned its service mix with this high-weight TCM-characteristic indicator.

##### 3.2.1.2 The 2024 step-change

In sharp contrast to the preceding pattern, TCM decoction piece usage rose abruptly to 17.74% in 2024, more than doubling the 2023 value (8.50%).

##### 3.2.1.3 Benchmarking and interpretation

Importantly, this 2024 value was not far above the annual national peer median of 16.49%, but rather slightly exceeded it by 1.25 percentage points. This suggests a threshold-crossing catch-up with strategic differentiation, rather than extreme gaming or simple homogeneity. In other words, the hospital appears to have moved from a prolonged below-median position to a just-above-median position on a highly weighted TCM indicator. This pattern is more consistent with selective catch-up under policy pressure than with indiscriminate overexpansion.

#### 3.2.2 The research surge

A similar but even more pronounced discontinuity was observed in the dimension of sustainable development.

##### 3.2.2.1 Historical context

Between 2019 and 2023, research funding per 100 health professionals fluctuated from 190,954 CNY to 324,513 CNY, with a sharp decline in 2022 (63,854 CNY), likely reflecting pandemic-era grant delays and the diversion of organizational attention toward COVID-19 response.

##### 3.2.2.2 The 2024 inflection point

In 2024, research funding rose sharply to 699,575 CNY, representing a 115.58% year-over-year increase. This magnitude of change clearly exceeded the institution's prior pattern of incremental adjustment.

##### 3.2.2.3 Strategic interpretation

Compared with the TCM decoction indicator, which mainly showed threshold-crossing catch-up, the research funding indicator demonstrated stronger strategic differentiation. Taken together, Figure 1 suggests that the hospital's 2024 response was selective rather than uniform. It caught up on a key TCM-identity metric while differentiating more aggressively in research-oriented investment.

### 3.3 Medical quality: stability amidst change

While the hospital intensified its efforts in TCM-characteristic performance and research development, its core medical quality indicators remained remarkably stable and competitive among peer institutions. This suggests that the hospital's selective strategic adaptation did not come at the expense of basic patient safety.

#### 3.3.1 Infection and complication control

The infection rate for Class I incisions remained at an extremely low level throughout the six-year period. This metric is critical for an orthopedic hospital, as implant infections, such as those in joint replacements or spinal fusions, can have catastrophic consequences. This indicator consistently outperformed the peer median (averaged at 0.15%) during the study period, demonstrating sustained adherence to sterile protocols. For surgical complications, except for a temporary increase in 2023 followed by a marked reduction in 2024, the rates remained extremely low and generally outperformed the national peer median.

#### 3.3.2 Antibiotic stewardship

The antibiotic use intensity (DDDs) ranged from 28.67 to 37.76, with a period average of 34.47. Although this exceeded the peer median of 29.13, it consistently remained below the national “red line” requirement (<40 DDDs) and declined consecutively in 2023 and 2024. These results suggest effective clinical governance and a move away from profit-driven antibiotic prescribing.

#### 3.3.3 Digital maturity

The EMR system application level remained stable at Level 4, meeting the national requirement for tertiary hospitals. This digital infrastructure supports data interoperability, real-time reporting, and performance monitoring, and provided an organizational foundation for the hospital's selective adaptation under the NPA.

### 3.4 Structural analysis: the crowding-out tendency

**Figure 2:**
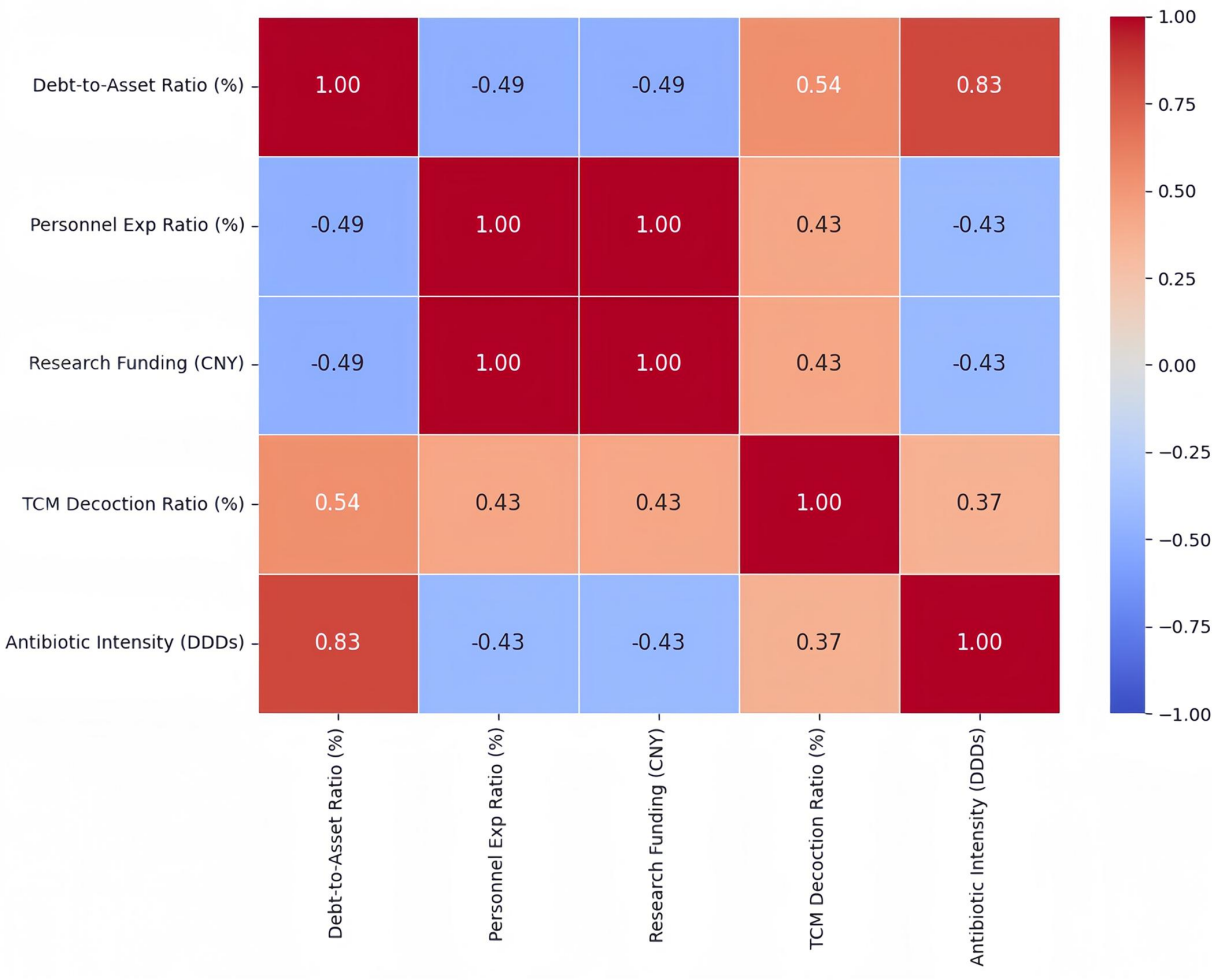
Spearman correlation matrix of key structural variables, illustrating internal resource allocation trade-offs and the crowding-out tendency in a small annual time series (2019-2024).

Despite the improvement in several output indicators, the input structure revealed persistent tensions in resource allocation. To visualize the internal structural relationships among key financial, strategic, and outcome variables, a Spearman correlation matrix was constructed (Figure 2), providing an integrated overview of the hospital's internal trade-offs.

Notably, the personnel expenditure ratio and research funding per 100 health professionals showed a perfect rank correlation $\left(r_{s}=1.00\right)$, reflecting identical temporal rank ordering across the six annual observations rather than implying a deterministic relationship.

#### 3.4.1 Financial leverage

Analysis of the hospital's capital structure revealed a persistently high degree of financial leverage. Across the study period, the debt-to-asset ratio fluctuated between 60.10% and 62.33%. This level was substantially higher than the national peer median (averaged at 36.92%), exceeding the benchmark by more than 20 percentage points. These findings suggest sustained reliance on debt financing to support modernization and institutional expansion. However, such leverage also implies greater financial vulnerability, particularly under conditions of reimbursement pressure and uncertain revenue growth.

#### 3.4.2 Human capital investment

Human capital investment analysis revealed a structural deviation from industry norms. The personnel expenditure ratio averaged 33.87% during the study period and reached 35.80% in 2024. Even with this increase, the level remained substantially below the national peer median of approximately 44%. These data reflect a persistent underinvestment in direct staff compensation compared to peer institutions. While the hospital appears to be strong in physical and organizational infrastructure, it is relatively austere in terms of actual employee payment. This may be regarded as a symptom of capital-labor substitution under debt constraints.

#### 3.4.3 Spearman analysis of the structural trade-off

**Figure 3:**
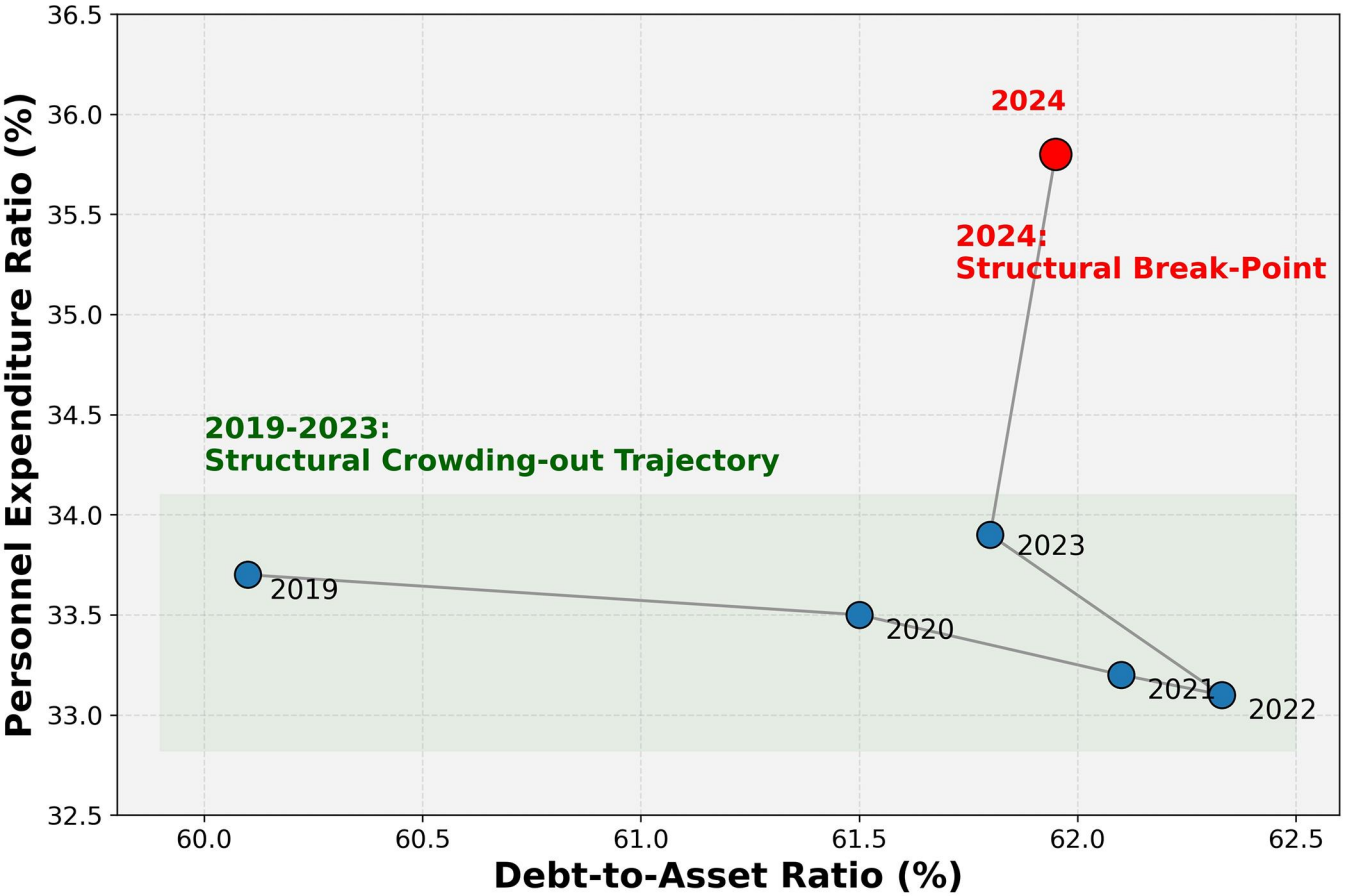
Annual trajectory of debt-to-asset ratio versus personnel expenditure ratio (2019-2024), illustrating a moderate inverse association and a 2024 break point in the earlier pattern.

Spearman rank analysis indicated a moderate inverse association between the debt-to-asset ratio and the personnel expenditure ratio $\left(r_{s}=-0.486,\;p=0.329\right)$ (Figure 3). Given the limited number of annual observations, this result should be interpreted as suggesting a crowding-out tendency rather than establishing a statistically definitive law.

The year-by-year trajectory in Figure 3 provides a more nuanced perspective than a single summary coefficient. From 2019 to 2023, the personnel expenditure ratio remained compressed at approximately 33%–34% despite persistently high leverage. In 2024, however, personnel expenditure rose to 35.80% while debt remained high at 61.95%, introducing a visible break point rather than reinforcing the earlier inverse pattern. This pattern suggests that the hospital may have temporarily suppressed wage growth during the earlier phase of debt-driven development, but that such suppression became increasingly difficult to sustain in the context of post-pandemic recovery and research-oriented transformation.

**Figure 4:**
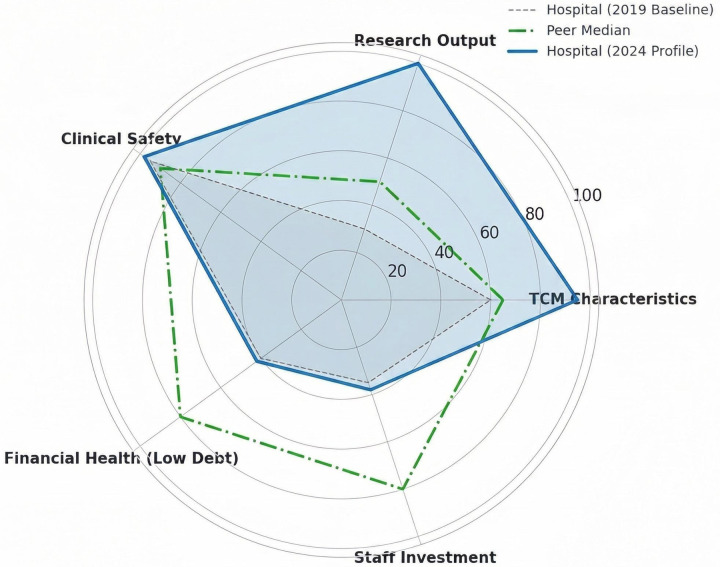
Radar chart comparing the hospital's 2019 baseline profile, 2024 profile, and peer median across TCM characteristics, research output, clinical safety, financial health, and staff investment.

To further integrate the findings at the strategic level, Figure 4 presents a radar chart comparing the hospital's baseline configuration (2019), its 2024 profile, and the national peer median. The chart highlights asymmetric growth in research output and TCM-characteristic performance relative to the peer benchmark, together with stable clinical safety, while also emphasizing persistent weaknesses in financial health and workforce investment.

### 3.5 Grey relational analysis results

To understand the drivers of overall hospital development (discharged patient volume), the study ranked the relational grades (γ) of key indicators based on the GRA calculations (Table 4).

**Table 4:** Grey relational grades of key indicators (ranked by influence).

Rank	Indicator Dimension	Specific Indicator	Relational Grade	Interpretation
1	Sustainability	Research funding per 100 health professionals	0.884	Primary driver
2	TCM Characteristics	Percentage of outpatient TCM non-drug therapy	0.862	Core strategy
3	Quality	Class I incision infection rate	0.795	Maintenance factor
4	Efficiency	Personnel expenditure ratio	0.612	Weaker influence
5	Efficiency	Debt-to-asset ratio	0.543	Weaker influence

Weaker influence: indicating the factor is likely influenced by other variables or has a less direct impact.

Among the drivers, research and TCM characteristics were the most significant (γ>0.85). This implies that the hospital's growth model was primarily driven by technological differentiation (specifically the outpatient TCM non-drug therapies with prominent features and visible benefits for patients' welfare) and academic expansion (tied to higher specialty rankings and perceived reputation by the public). Conversely, efficiency indicators (financial and personnel) showed relatively low relational grades (γ<0.65), suggesting that cost-efficiency and compensation structure improvement were not the dominant drivers of organizational growth during this period. Considering Figure 1, these findings support an interpretation of selective strategic adaptation, that is, the hospital strengthened high-weight identity and prestige indicators but did not achieve commensurate improvement in the underlying balance between financial resilience and human capital investment.

## 4 Discussion

### 4.1 Interpreting selective strategic adaptation under layered reform pressures

While this is a single-center study, the longitudinal design spanning the full duration of the NPA reform provides an in-depth perspective on the dynamic adaptation process that cross-sectional, multi-center studies cannot capture. The findings support the view that China's National Performance Appraisal is not merely a performance monitoring tool but an important institutional mechanism for shaping organizational behavior. However, the 2024 acceleration should not be attributed to NPA pressure alone. Rather, it is more plausibly interpreted within a layered reform environment, in which post-pandemic operational normalization, DRG (Diagnosis-Related Group)/DIP (Diagnosis-Intervention Packet) payment reform, and NPA score incentives were acting simultaneously. This layered interpretation is also consistent with recent scholarship portraying China's current public hospital reform as a high-quality development phase characterized by the simultaneous reconfiguration of governance, financing, payment, and evaluation mechanisms (29–31).

From an institutional theory perspective, the 2024 pattern is better understood as selective strategic adaptation rather than undifferentiated strategic isomorphism (32–34). The TCM decoction indicator reflects a threshold-crossing catch-up process. The hospital moved from persistently below the annual peer median to slightly above it in 2024, which is more consistent with legitimacy-seeking catch-up than with extreme divergence. By contrast, the research funding indicator reflects a stronger form of strategic differentiation, suggesting that the hospital's response was selective rather than uniform.

This pattern also highlights the risk of high-stakes metric steering, often described by Goodhart's Law: when a specific indicator becomes a target, it may no longer function as a neutral indicator (59). The rise in TCM decoction use to 17.74% therefore requires cautious interpretation. It may reflect genuine strategic reorientation, but it may also reflect the hospital's rational response to overlapping institutional and financial incentives. Although the continued stability of core quality indicators such as surgical infection rates and antibiotic use intensity suggests that baseline patient safety was not compromised, the risk of metric fixation remains a professional concern (60). Future qualitative research is needed to determine whether the observed increase in TCM-characteristic services primarily reflected clinical appropriateness, administrative targeting, or both.

### 4.2 Resource dependence, human capital, and the emergence of structural trade-offs

The analysis further suggests that the hospital's strategic realignment was accompanied by a reconfiguration of its internal resource structure. Grey relational analysis identified research output and TCM-characteristic indicators as the most important drivers of overall development, whereas conventional efficiency indicators were less influential. This pattern aligns with resource dependence theory, which predicts that organizations rearrange internal priorities to secure externally controlled resources, in this case NPA rankings, subsidies, policy legitimacy, and reputational benefits (35–37).

At the same time, the Spearman analysis indicates a moderate inverse association between financial leverage and personnel expenditure. This result should be interpreted as suggesting a crowding-out tendency rather than establishing a definitive causal law. Even so, the pattern remains economically meaningful. When a hospital relies on debt-financed infrastructure expansion, the need to service hard-asset investment may narrow the fiscal space available for workforce compensation, especially when operating margins are simultaneously constrained by case-based payment reform (61–66, 79).

A comparative perspective helps clarify the contribution of this study. Relevant literature showed in Ethiopian public and private hospital settings that tight regulation, limited strategic human resource management autonomy, and constrained flexibility in pay and working conditions can undermine workforce outcomes and organizational performance (67–70). Our study extends that broader insight into a distinct Chinese TCM-hospital context, where workforce pressure arises not only from generic public-sector resource scarcity, but from the coexistence of capital-intensive modernization demands, labor-intensive TCM-characteristic mandates, and reimbursement reform. In this sense, the present study is not merely about financial leverage; it is about how a specific institutional configuration reshapes the labor-capital balance inside a tertiary public TCM hospital. Beyond the Ethiopian comparison, recent cross-country policy analyses further suggest that China's current reform trajectory is distinctive in the extent to which public welfare goals, performance appraisal, payment reform, and digital governance are being advanced in parallel, making the TCM hospital setting especially informative for studying how multiple institutional pressures interact inside one organization (29, 30).

This interpretation is also consistent with the trajectory shown in Figure 3. From 2019 to 2023, personnel expenditure remained compressed at approximately 33%–34% despite persistently high leverage, whereas 2024 introduced a visible break point in the earlier pattern. Rather than proving a stable linear rule, the data suggest a structurally strained equilibrium that may have become increasingly difficult to maintain.

From the perspective of Baumol's cost disease, the findings are better interpreted dynamically than statically (71, 72). Instead of refuting the tendency for labor costs to rise in labor-intensive sectors, the data suggest that this pressure may have been temporarily offset during 2019–2023 by debt-financed development and capital prioritization. The simultaneous increase in both debt and personnel spending in 2024 indicates that the earlier pattern of wage compression may have become hard to sustain.

The policy relevance of the crowding-out finding is strengthened. If debt is used to fund modernization and hard-asset expansion at the same time that DRG/DIP reform compresses margins on routine services, the burden of adjustment may shift onto labor expenditure. In such a scenario, the crowding-out tendency is not merely an internal budgeting issue but a predictable consequence of layered incentives that reward visible output while under-protecting workforce sustainability.

### 4.3 Implications for TCM hospitals under payment reform

The observed increase in TCM decoction and non-drug therapy usage should be interpreted not only within the NPA framework, but also within the broader context of China's ongoing DRG/DIP payment reform (73, 79). Recent literature suggests that case-based payment reform can alter provider behavior through cost shifting, patient selection, coding incentives, and service substitution. In the TCM context, this issue is especially important because payment reform interacts with a dual-track service model in which traditional medicine and conventional medicine coexist within the same institutional setting (63–66, 74).

This point is particularly salient for TCM hospitals. Recent studies have shown that DRG reform in TCM hospitals may produce both cost-control gains and unintended side effects, while policy documents and empirical studies increasingly recognize the need to incorporate TCM specialty therapies, Chinese herbal medicines, and TCM-oriented coefficients into DRG/DIP design (63). At the same time, evidence from a Chinese integrative medicine hospital under DIP reform indicates that conventional medicine expenditures decreased while traditional medicine expenditures increased, suggesting a service substitution effect rather than a purely cultural revival (74). In this light, the 2024 increase in TCM-characteristic services in our study is more plausibly interpreted as a dual strategy: securing NPA scores and administrative legitimacy on the one hand, while preserving service revenue and margin flexibility under case-based payment constraints on the other.

This interpretation also helps explain why the 2024 shift should not be described as an isolated policy-compliance event. In an environment where surgical and other standardized inpatient services are increasingly constrained by DRG/DIP payment ceilings, as well as by procurement-side cost-containment reforms such as national volume-based procurement, hospitals may rationally expand TCM-characteristic services, which are institutionally protected, symbolically valued, and, in some cases, comparatively more adaptable within the payment architecture (47, 75, 79). Thus, the 2024 acceleration appears to reflect not only cultural repositioning, but also financial hedging behavior under layered reform pressure.

The rise in research funding can be interpreted in a similar way. While the surge certainly reflects the hospital's pursuit of academic prestige and its response to NPA sustainability indicators, it may also represent a diversification strategy under tighter clinical operating margins. Research grants, special projects, and academic reputation become increasingly attractive when routine revenue sources are constrained. Accordingly, the 2024 combination of intensified TCM-characteristic services and intensified research investment is better understood as a coordinated adaptation to both score-based governance and payment-based revenue compression.

### 4.4 Sustainability risks and post-pandemic resilience

The combination of high financial leverage and relatively low personnel spending signals a potential sustainability challenge. Healthcare delivery remains inherently labor-intensive, particularly in specialty care, teaching, and research settings (76). Consequently, chronic underinvestment in human capital risks eroding staff motivation and weakening long-term retention. As the hospital shifts toward a research-oriented model that requires higher-level talent, the imbalance between capital investment and salary competitiveness may constrain the very innovative capacity the institution seeks to build (77). This concern is also consistent with Figure 2, in which the personnel expenditure ratio and research funding per 100 health professionals showed identical temporal rank ordering across the six annual observations $\left(r_{s}=1.00\right)$. Rather than implying a deterministic relationship, this pattern suggests that the hospital's research-oriented development remained closely tied to the trajectory of human capital investment, reinforcing the argument that academic expansion cannot be sustained if personnel support remains structurally constrained.

Despite these structural risks, the 2024 data also demonstrate post-pandemic resilience. The sharp increase in scientific research funding and the recovery of high-weight TCM indicators suggest that this hospital retained significant adaptive capacity after pandemic-era disruption. What remains uncertain, however, is whether this recovery represents the beginning of a sustainable new equilibrium or a short-term strategic response achieved at considerable internal cost. This question is especially important for lower-tier TCM hospitals, which may not possess the same access to credit, grants, or political leverage as a National Regional Medical Center.

### 4.5 Study limitations

This study is subject to several limitations inherent in its design and data scope, which should be considered when interpreting the findings.

First, in terms of generalizability, this is a single-center study focusing on a Grade-A tertiary TCM orthopedic hospital that also serves as a National Regional Medical Center. Therefore, the pattern of debt-supported modernization and selective strategic adaptation observed here may not be applicable to smaller municipal or county-level TCM hospitals that lack comparable credit access, grant capacity, or political leverage. The strategic shift toward research intensity and stronger TCM-characteristic performance may require a level of institutional resource flexibility that is unavailable in lower-tier settings.

Second, this study is limited by its exclusive reliance on quantitative administrative data. While the 2024 step-change in selected indicators provides evidence of selective strategic adaptation, the lack of qualitative data (e.g., interviews with hospital leadership and department heads) constrains deeper interpretation of the internal decision-making processes (78). Consequently, it remains unclear to what extent the increase in TCM decoction use was driven by clinical necessity, administrative targeting, financial hedging under DRG/DIP reform, or a combination of these factors. Moreover, this study analyzed organizational output indicators rather than patient-level health outcomes; therefore, whether the strengthened TCM-characteristic performance translated into improved clinical effectiveness remains to be examined. The reported values for medical quality indicators, especially incision infection and surgical complication rates, should also be interpreted with caution because they rely on EMR summary page data (11, 49). Although routine internal data quality control was performed, these indicators may still be influenced by reporting constraints and differences in clinical interpretation. Future research should combine administrative, qualitative, and patient-level clinical data to reduce the limitations inherent in summary-level organizational analysis.

Third, regarding causal inference, neither Spearman correlation analysis nor grey relational analysis can establish causation in a six-year observational series. The behavioral changes observed in this hospital occurred within a multifaceted reform environment that included NPA implementation, DRG/DIP payment reform, and post-pandemic regional recovery (29, 31, 79). Accordingly, the hospital's increased emphasis on TCM-characteristic services and research investment should be interpreted as potentially shaped by overlapping institutional and financial incentives rather than by the NPA alone. Future multi-center comparative studies, ideally using quasi-experimental designs such as difference-in-differences, should examine whether similar adaptation patterns emerge across different hospital tiers, ownership structures, and payment-reform environments.

## 5 Conclusion

This study used six years (2019–2024) of longitudinal performance data to examine how a tertiary public TCM hospital adapted to China's National Performance Appraisal system under post-pandemic recovery and layered reform pressures. The findings suggest that the hospital did not respond through uniform improvement across all dimensions, but through selective strategic adaptation. It strengthened high-weight TCM-characteristic and research indicators while maintaining stable core clinical safety indicators. In particular, the 2024 increase in TCM decoction piece usage is better interpreted as a step-change and threshold-crossing catch-up relative to the annual peer median, rather than as a classic J-curve or a simple case of homogeneity.

At the same time, the study reveals a critical structural tension. The hospital's apparent performance gains were accompanied by persistently high leverage and chronically constrained personnel expenditure. Although the Spearman analysis supports only a moderate inverse association rather than a statistically definitive law, the overall pattern suggests a crowding-out tendency in which debt-reliant modernization may narrow the fiscal space available for human capital investment. In this sense, the apparent success of the NPA in driving TCM-characteristic and research metrics appears financially precarious, because it has been achieved under a capital structure characterized by sustained high leverage rather than balanced organizational strengthening.

These findings imply that performance governance may generate visible indicator gains while preserving underlying vulnerabilities in workforce sustainability and financial resilience. To promote genuinely sustainable high-quality development, the appraisal framework should move beyond rewarding output indicators alone and incorporate stronger safeguards for organizational balance. Specifically, the NPA scoring system could introduce a debt-to-service capacity or leverage-risk indicator as a negative adjustment factor to discourage overextended debt-fueled expansion. In parallel, eligibility for NPA-recognized research funding and other development-oriented rewards could be tied to demonstrated improvement in personnel expenditure ratios, so that human capital keeps pace with physical and academic expansion.

For policymakers, the central implication is that high-quality development cannot be assessed solely through visible output indicators. For hospital managers, the key challenge is not merely to improve appraisal scores, but to translate short-term performance improvements into a more balanced development model in which TCM identity, academic growth, financial resilience, and workforce sustainability advance together.

## Data Availability

The original contributions presented in the study are included in the article/Supplementary Material, further inquiries can be directed to the corresponding author/s.
